# Supplementary material for: Novel competition test for food rewards reveals stable dominance status in adult male rats
Source: Sci Rep. 2021 Jul 16;11:14599. doi: 10.1038/s41598-021-93818-0 (PMC8285491; doi:10.1038/s41598-021-93818-0)
Supplement: Supplementary file 1 — Supplementary Information 1. [file 41598_2021_93818_MOESM1_ESM.docx]

**Supplemental Figures**

**Novel Competition test for food rewards reveals stable dominance status in adult male rats**

Diana F Costa, Marta A Moita and Cristina Márquez


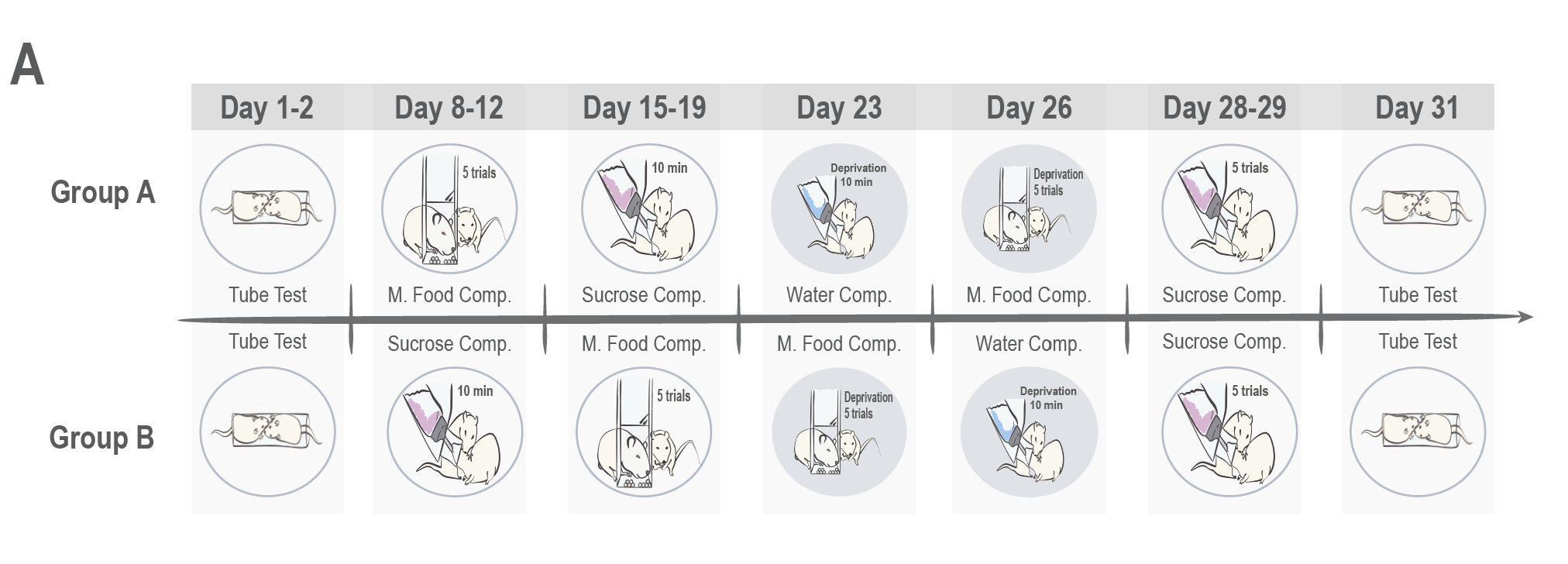


**Supplemental Figure 1: Timeline of experimental design.** After handling, two independent groups of animals were created where the order of the tests was counterbalanced. The order of the test is provided in the cartoon, and days indicate when they were performed. Note that in the case of the first evaluation of the Tube test, the modified Food Competition test without deprivation and the Sucrose Competition with continuous access to the bottle (10 minutes), these days include also the habituation sessions. All animals were tested in all competition tasks with inter-tasks intervals ranging between 2 and 6 days. Sucrose competition with intermittent access to the bottle (5 trials) was included towards the end of the experiment, after realization of the low amounts of drinking performed in the sucrose test with the continuous access to the bottle configuration. Shaded circles in the competition tasks at days 23 and 26 indicate that they were performed under deprivation states.

**Supplemental Figure 2: Identification of moments with highest conflict in the Water Competition tests and lack of correlation with Dominance Index (DI) in any other test.** (**A**) Graphical representation of dynamics of consumption (red, left axis) and pushing behaviors (green, right axis) and selection of the bout with highest conflict (shaded red rectangle) in three example pairs of animals during Water Competition test. Drinking and pushing raw data was transformed into modified cumulative plots and smoothed using convolution with a Gaussian filter of 30ms standard deviation. In this plots the direction of the cumulative graph indicated which animal was drinking or pushing over the session, either animal A or B. In this way, increases in this cumulative plot would indicate that animal A would be drinking, decreases that animal B would drink, and flat stable lines that no animal was drinking. The same applied for pushing data (in green). For example, for the example pair 1 animal B would start drinking while animal A was pushing for around 100 seconds, then they would alternate for a brief period of time, followed by another alternation, where a long bout of drinking was performed again by animal B while animal A continued pushing. After that, no significant pushing was performed by any of the animals, and although some alternations in drinking would be observed, now animal A would take over and drink more. In the different example graphs we can observe that dynamics between pairs are different over time but that animals mostly alternate in their drinking times, and pushing behavior decreases around half of the session. In order to select the bouts of highest conflict, we first defined epochs of consumption and pushing displayed by the pair by identifying the turning points that marks the moments when significant changes in the behavior occurs ( $x\left[ n+1 \right]-x\left[ n \right]<0$ ). For each epoch we calculated the duration of both consumption and pushing behaviors and selected the epoch with the highest value of both consumption and time pushing the partner from the water dispenser. (**B**) No significant correlation was observed between Dominance Indexes (DI) of Water Competition when calculated with the drinking duration in the whole session, nor in the first min or the two first minutes, nor when taking into account the moment when animals started drinking. We then identified the epochs with longest drinking, as they could be not necessarily in the early moments of the test, but no correlation was observed with other behavioral tasks either. We then selected the epochs with higher conflict (see A for representative examples of the identification of the epochs), but again, no correlation was observed. Values in the correlation matrix correspond to Pearson correlation r values. #p<0.10.


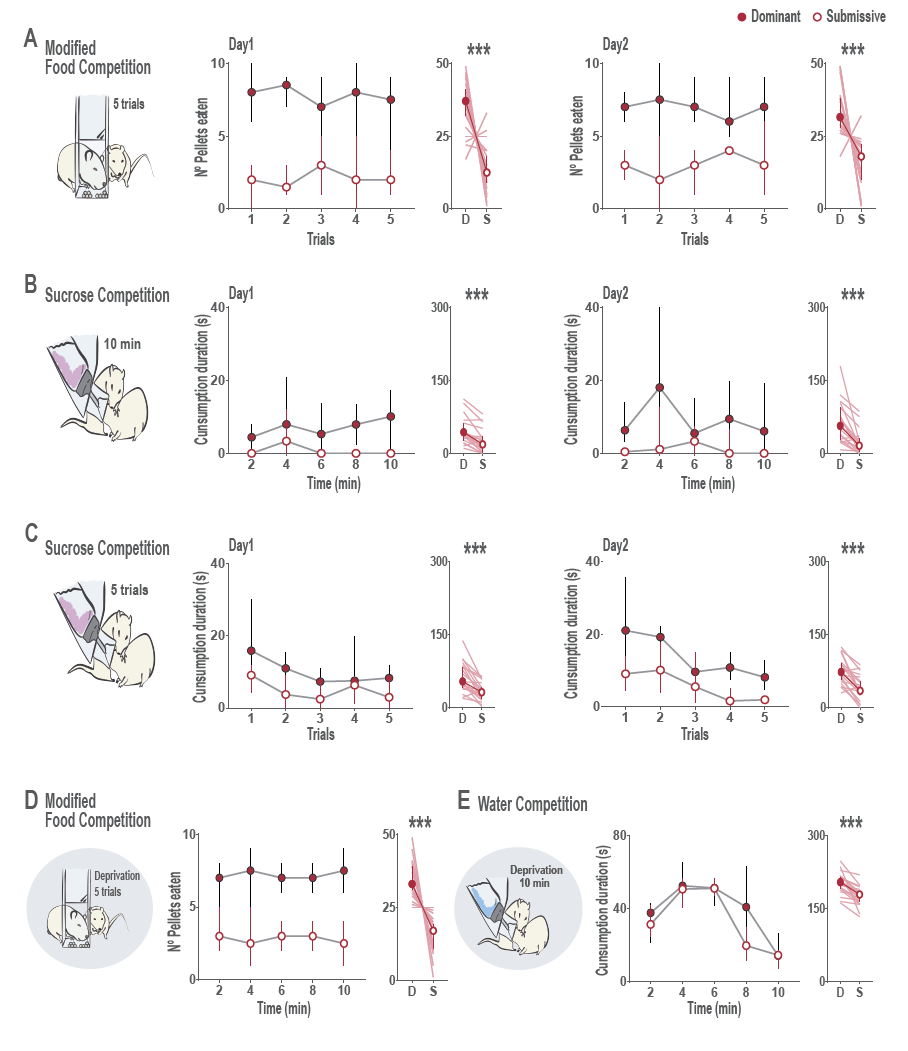


**Supplemental Figure 3: Social hierarchy measures are stable across time.** Consumption of resources is plotted over time or trials within a session and across days. In all cases the total consumption of all testing days was taken as the criteria to define dominant and submissive animals. In those tasks with a trial structure, results for each day are first presented by trials and then as the average of consumption in that day. For the sucrose competition with continuous access and the water competition, results are first presented in 2 min blocks and then the average consumption of that day. Statistics evaluating differences between dominant and submissive animals were performed in the average consumption of a day (**A**) consumption in the modified Food Competition on each of the two days of testing when the criteria to define hierarchy was the total consumption of the two days. Dominant and submissive animals clearly differed in their consumption across time. (**B**) Similar for the Sucrose Competition with continuous access to the bottle. Note the low levels of consumption, especially in day 1. (**C**) Similar for the Sucrose Competition with intermittent access, (**D**) the modified Food Competition under deprivation and (**E**) the Water Competition. Median, 95% CI and individual values for all animals are represented. ***p<0.001 after non parametric Wilcoxon test.


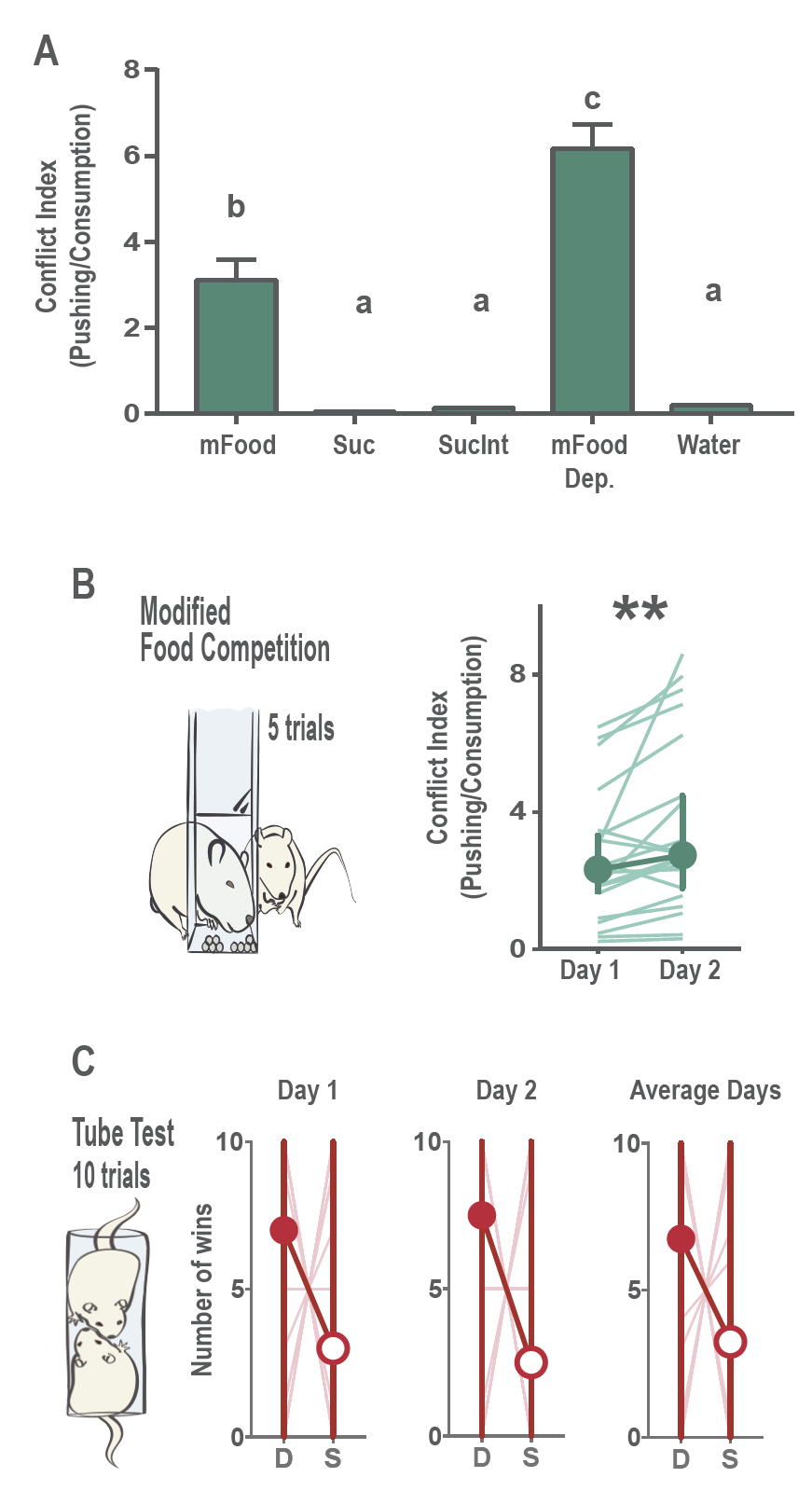


**Supplemental Figure 4: Food Competition tests reflect higher conflict**. (**A**) We calculated a conflict index for each test that involved consumption of resources by dividing the duration of pushing performed by the time the spent consuming (as the duration to consume 1 pellet would be few milliseconds in the case of the Food Competition tests, we considered the latency to eat the 10 pellets available in each trial). Due to the short duration of pellet availability and high pushing levels observed in the Food Competition tests, these tests showed higher conflict indexes than the sucrose tests or water competition (F(4, 98)=64.805 p<0.0001 followed by Tukey posthoc), which was more marked when animals were under deprivation. Average and SEM are represented, and letters denote statistically significant differences between behavioral tests after one-way ANOVA with Tukey posthoc comparisons. (**B**) Conflict index was higher in the second day of testing in the modified Food Competition test (paired t-test t(19)=-2.985 p=0.008). Median, 95% CI and individual values for all animals are represented. ** p>0.01. (**C**) Dominant animals defined according to their behavior in the second day of modified Food Competition test did not differ in the amount of winnings in the tube test in either of the days tested nor when the average winnings of the two days were considered (Wilcoxon signed-ranks for TT Day1: z=-0.774, p=0,44; TT Day2: z=-0.503, p=0,62; TT AVG Days = z=-0.699, p=0,49). Median, 95% CI and individual values for all animals are represented
