# Supplementary material for: Novel competition test for food rewards reveals stable dominance status in adult male rats
Source: Sci Rep. 2021 Jul 16;11:14599. doi: 10.1038/s41598-021-93818-0 (PMC8285491; doi:10.1038/s41598-021-93818-0)
Supplement: Supplementary file 2 — Supplementary Information 2. [file 41598_2021_93818_MOESM2_ESM.docx]

**Multimedia Legends**

MOVIE 1- IMAGE TO APPEAR ON THE PRINT ISSUE


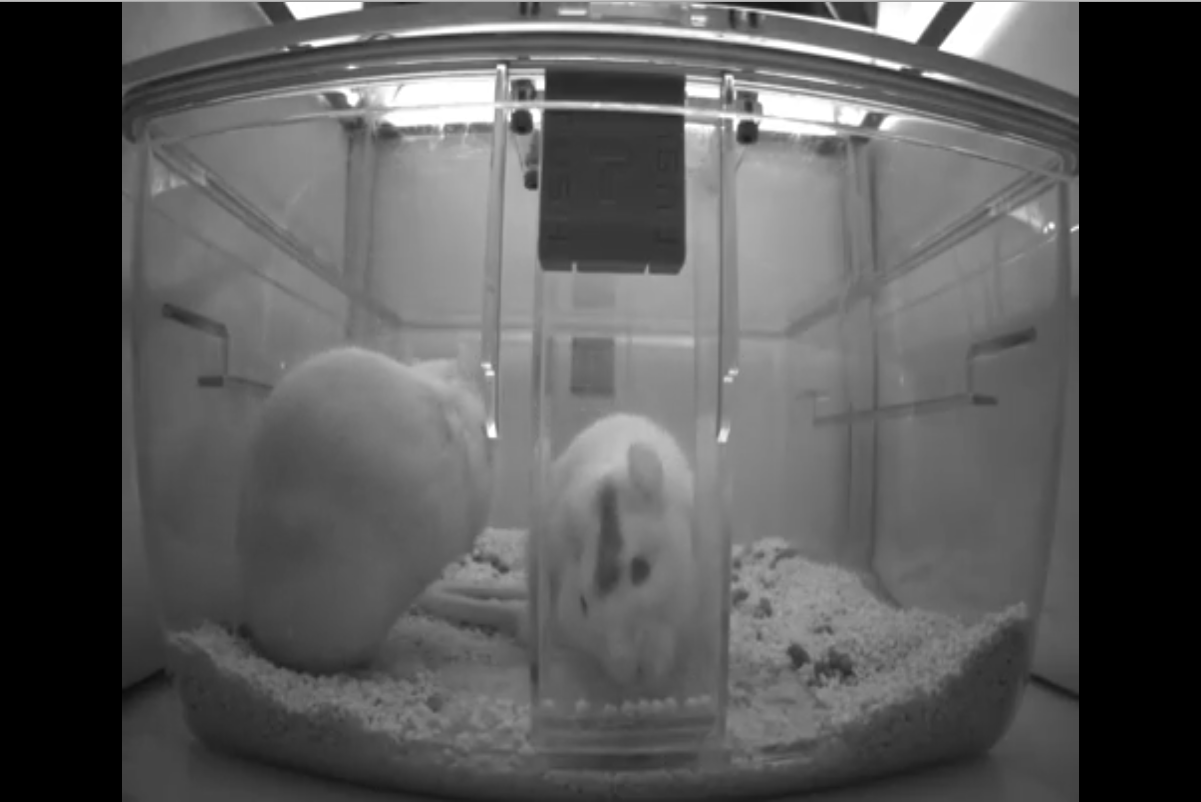


**Movie 1- Representative video of a trial in the modified Food Competition test.** A pair of non-food-deprived cage mates compete for the access to a feeder placed in the home cage containing palatable pellets. The structure of the feeder allows access to only one animal at the time. During inter-trial interval, a transparent partition prevents access to the rewards. Once the partition is removed, the trial starts, and animals are allowed to consume the food pellets. Multiple bouts of successful and unsuccessful pushing behaviors can be observed in order to gain access to the food.
